# Disposable Puff Bar Electronic Cigarettes: Chemical Composition and Toxicity of E-liquids and a Synthetic Coolant

**DOI:** 10.1021/acs.chemrestox.1c00423

**Published:** 2022-07-18

**Authors:** Esther E. Omaiye, Wentai Luo, Kevin J. McWhirter, James F. Pankow, Prue Talbot

**Affiliations:** † Environmental Toxicology Graduate Program, University of California Riverside, Riverside, California 92521, United States; ‡ Department of Civil and Environmental Engineering, Portland State University, Portland, Oregon 97201, United States; § Department of Chemistry, Portland State University, Portland, Oregon 97201, United States; ∥ Department of Molecular, Cell, and Systems Biology, University of California Riverside, Riverside, California 92521, United States

## Abstract

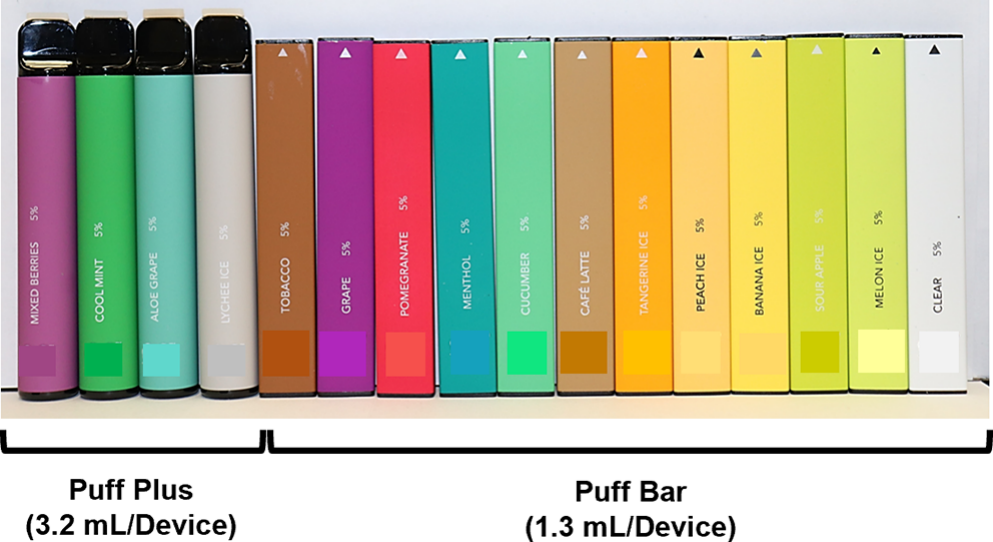

The popularity of disposable fourth-generation electronic cigarettes (ECs) among young adults and adolescents has been increasing since the ban on flavored cartridge EC products such as JUUL. Although the constituents and toxicity of some cartridge-based fourth-generation ECs, such as JUUL, have been studied, limited data exist for other disposable ECs such as Puff. The purpose of this study was to determine flavor chemicals, synthetic coolants, and nicotine concentrations in 16 disposable Puff devices, evaluate the cytotoxicity of the different flavors from the Puff brand using in vitro assays, and investigate the health risks of synthetic coolants in EC products. Gas chromatography/mass spectrometry was used to identify and quantify chemicals in Puff EC fluids. One hundred and twenty-six flavor chemicals were identified in Puff fluids, and 16 were >1 mg/mL. WS-23 (2-isopropyl-*N*,2,3-trimethylbutyramide) was present in all products, and concentrations ranged from 0.8 to 45.1 mg/mL. WS-3 (*N*-ethyl-*p*-menthane-3-carboxamide) concentrations ranged from 1.5 to 16.4 mg/mL in 6/16 products. Nicotine concentrations ranged from 40.6 to 52.4 (average 44.8 mg/mL). All unvaped fluids were cytotoxic at dilutions between 0.1 and 10% in the MTT and neutral red uptake assays when tested with BEAS-2B lung epithelial cells. The cytotoxicity of Puff fluids was highly correlated with total chemical concentrations, nicotine, WS-23, both synthetic coolants, and synthetic coolants plus ethyl maltol. Lower concentrations of WS-23 than those in the fluids adversely affected cell growth and morphology. Concentrations of synthetic coolants exceeded levels used in consumer products. The margin of exposure data showed that WS-3 and WS-23 concentrations were high enough in Puff products to present a health hazard. Our study demonstrates that disposable Puff ECs have high levels of cytotoxic chemicals. The data support the regulation of flavor chemicals and synthetic coolants in ECs to limit potentially harmful health effects.

## Introduction

Electronic cigarettes (ECs), which contain nicotine, solvents, and flavor chemicals, continue to evolve and grow in popularity, especially among young adults.^1−6^ The popularity of fourth-generation EC products and their disposable spinoffs, especially among young users, has been attributed to flavored and “icy” fluids, usability, and device features that facilitate stealth use.^7−12^ EC fluids and aerosols generated from multiple devices contain higher concentrations of chemicals than used in other consumer products, such as foods, cosmetics, and medicines.^13−15^ ECs and their constituents are cytotoxic to cells, induce inflammatory responses, increase oxidative stress, cause cellular senescence, and negatively affect cell membrane channel potentials.^16−23^ Despite concern over the use of flavor chemicals in ECs, the chemicals used in EC fluids continue to change and are largely unregulated. Even though JUUL dominates the EC market with 63% of current sales,^24,25^ projections show that disposables, such as Puff Bar, are likely to continue to increase their sales through 2028.^26^

The technology used by manufacturers of fourth-generation ECs, such as JUUL and Puff Bar, is innovative. Nicotine is combined with an acid(s) to reduce the amount of free-base nicotine, making the resulting aerosol less harsh. The use of acids allows manufacturers to increase nicotine concentrations (e.g., 61 mg/mL in JUUL)^27,28^ while making it less harsh to users,^29−31^ thereby increasing the likelihood of addiction. To reduce sales of JUUL to young users, the Food and Drug Administration (FDA) enacted a ban on cartridge-based flavored EC pods in 2020.^32^ Consumers and suppliers quickly discovered a loophole in the ban, which did not cover “disposable” flavored EC products, such as Puff ECs.^33,34^ The market for disposable pods continues to grow, with dozens of products offered by multiple purveyors.^35,36^

Although Puff ECs are the most widely used of the fourth-generation disposable products, very little is known about their fluids’ chemical composition and toxicity. The purpose of our study was to (1) identify and quantify nicotine, flavor chemicals, and synthetic coolants in Puff fluids, (2) determine the toxicity of the Puff fluids and WS-23 in multiple assays, (3) evaluate the transfer efficiency of synthetic coolants to aerosols, and (4) perform (margin of exposure) MOE risk assessment analysis on synthetic coolants in Puff products.

## Materials and Methods

### Materials

Isopropyl alcohol (IPA), Dulbecco’s phosphate-buffered saline (DPBS), dimethyl sulfoxide (DMSO), ethanol (EtOH), and acetic acid were purchased from Fisher Scientific (Chino, CA). Analytical grade WS-3 (*N*-ethyl-*p*-menthane-3-carboxamide) (CAS # 39711-79-0; catalog #E0796; Lot: SYXVH-SP) and WS-23 (2-isopropyl-*N*,2,3-trimethylbutyramide) (CAS # 51115-67-4; catalog #I0729; Lot: LTNPJ-DP) both >98% pure were purchased from Tokyo Chemical Industry Co. LTD. (Portland, OR). BEAS-2B cells were obtained from American Type Cell Culture (ATCC, Manassas, VA). Bronchial epithelial basal medium (BEBM) and supplements were purchased from Lonza (Walkersville, MD). Collagen (30 mg/mL), bovine serum albumin (BSA, 10 mg/mL), fibronectin (10 mg/mL), poly-vinyl-pyrrolidone (PVP), MTT reagent (3-(4,5-dimethylthiazol-2-yl)-2,5-diphenyltetrazolium bromide), NRU dye (neutral red uptake dye), Tris-HCl, Tris-base, lithium lactate, tetrazolium salt (INT), phenazine methosulfate (PMS), and β-nicotinamide adenine dinucleotide (NAD) sodium salt were purchased from Sigma-Aldrich (St Louis, MO).

### Sample Acquisition

Sixteen disposable Puff EC devices were purchased from vape shops in Los Angeles, CA, and Riverside, CA, in 2020. Twelve Puff Bar flavors (“Tobacco,” “Grape,” “Pomegranate,” “Cucumber,” “Café Latte,” “Tangerine Ice,” “Peach Ice,” “Banana Ice,” “Sour Apple,” “Melon Ice,” “Menthol,” and “no flavor” (“Clear”)) were labeled to contain 1.3 mL of fluids and advertised to deliver 300 puffs/device. Four Puff Plus flavors (“Mixed Berries,” “Aloe Grape,” “Cool Mint,” and “Lychee Ice”) were labeled to contain 3.2 mL of fluids and advertised to deliver 800 puffs/device. All devices were inventoried, stored in the dark at room temperature, and analyzed within 2–3 weeks of purchase.

Authentic standards of both WS-3 and WS-23 were dissolved in propylene glycol (PG, 80%) and distilled water (<20%) to simulate lab-made refill fluids. A PG control blank was prepared with 80% PG and 20% distilled water.

### Aerosol Production and Capture Using an Impinger Method

The transfer efficiency of synthetic coolants from lab-made fluids into the aerosols was evaluated using a fourth-generation Baton V2 open pod system equipped with a 350 mAh rechargeable battery, a 1.5 mL refillable pod, and a 1.6 Ω coil that produces an aerosol at 3.7 V/8.6 W. Refillable pods were filled with lab-made fluids and preconditioned by taking three puffs before making aerosol solutions. The generated aerosol was bubbled through and captured in IPA for chemical analysis. The WS-3 and WS-23 aerosol materials captured in IPA (referred to as “aerosol”) were collected at room temperature in two tandem 125 mL impingers, each containing 25 mL of IPA. The Baton V2 pod system was connected to a Cole-Parmer Masterflex L/S peristaltic pump and was puffed using a 4.3 s puff duration,^21^ interpuff intervals of 60 s, and an airflow rate of 10–13 mL/s. To reduce the likelihood of “dry puffing,” the fluid level was monitored, and the device was not vaped beyond 3/4 of the pod. The pods were weighed before and after aerosol production to collect at least 10 mg for gas chromatography/mass spectrometry (GC/MS) analysis. Aerosol solutions were stored at −20 °C until shipped to Portland State University for analysis.

### Gas Chromatography/Mass Spectrometry

Puff ECs containing fluid-saturated wicks were dissected to expose the atomizers. The fluid-saturated wicks were centrifuged in Qiagen MinElute spin columns (Valencia, CA) at 3000 rpm for 3 min to separate the fluid from the wick. The extracted fluid was analyzed using previously described GC/MS methods.^28,37^ Each sample (50 μL) was dissolved in 0.95 mL of IPA and shipped overnight on ice to Portland State University, where they were analyzed on the day they were received. A 20 μL aliquot of internal standard solution (2000 ng/μL of 1,2,3-trichlorobenzene dissolved in IPA) was added to each diluted sample before analysis. Using internal-standard-based calibration procedures described elsewhere,^37^ analyses for 178 flavor-related target analytes, two synthetic coolants, and nicotine were performed with an Agilent 5975C GC/MS system (Santa Clara, CA). A Restek Rxi-624Sil MS column (Bellefonte, PA) was used (30 m long, 0.25 mm id, and 1.4 μm film thickness). A 1.0 μL aliquot of the diluted sample was injected into the GC with a 10:1 split. The injector temperature was 235 °C. The GC temperature program for analyses was 40 °C hold for 2 min, 10 °C/min to 100 °C, then 12 °C/min to 280 °C and hold for 8 min at 280 °C, and then 10 °C/min to 230 °C. The MS was operated in the electron impact ionization mode at 70 eV in the positive-ion mode. The ion source temperature was 220 °C, and the quadrupole temperature was 150 °C. The scan range was 34 to 400 amu. Each of the 181 (178 flavor chemicals, 2 synthetic coolants, and nicotine) target analytes was quantitated using the authentic standard material.

In October 2019, two synthetic coolants (WS-3 and WS-23) and triethyl citrate were added to our GC/MS target list, which is used to identify and quantify flavor chemicals. GC/MS data collected for multiple EC libraries from 2016 to September 2019 were re-evaluated to estimate the concentrations of synthetic coolants (WS-3 and WS-23) and triethyl citrate using the average response factors generated for them between October 2019 and December 2019.

### Human Bronchial Epithelial Cells (BEAS-2B)

Experiments were performed using BEAS-2B cells (passages 20–34), often used for toxicological testing. BEAS-2B cells exposed to menthol in submerged culture gave similar results to 3D EpiAirway exposed at the air-liquid interface^38^ and therefore represent a good cell type for initiating work on the synthetic coolants. BEAS-2B cells were cultured in bronchial epithelial growth medium (BEGM) supplemented with 2 mL of the bovine pituitary extract and 0.5 mL each of insulin, hydrocortisone, retinoic acid, transferrin, triiodothyronine, epinephrine, and human recombinant epidermal growth factor. Nunc T-25 tissue culture flasks were coated overnight with BEBM fortified with collagen (30 mg/mL), BSA (10 mg/mL), and fibronectin (10 mg/mL) before culturing. Cells were maintained at 30–90% confluence at 37 °C in a humidified incubator with 5% carbon dioxide. For subculturing, cells were harvested using DPBS for washing and incubated with 1.5 mL of 0.25% trypsin EDTA/DPBS and PVP for 3–4 min at 37 °C to allow detachment. Cells were counted using a hemocytometer and cultured in T-25 flasks at 75,000 cells/flask. The medium was replaced the next day and then every other day.

For in vitro assays, cells were cultured and harvested at 80–90% confluency, using protocols previously described.^15^ For the MTT, NRU, and LDH (lactate dehydrogenase) assays, cells were plated at 10,000 cells/well in precoated 96-well plates and allowed to attach overnight before a 24 h treatment. BEAS-2B cells were plated at 42,000 cells/well in precoated 24-well plates for the live-cell imaging experiments.

### Cytotoxicity and Cell Viability Assays

The effects of Puff fluids on the activity of mitochondrial reductase, neutral red uptake, and LDH release were evaluated. In the culture medium, serial dilutions of EC fluids (10, 3, 1, 0.3, 0.1, and 0.03%) were arranged in 96-well plates with negative controls (0%) placed next to the highest and lowest concentrations to check for avapor effect.^39^ BEAS-2B cells were seeded and allowed to attach for 24 h. Cells were exposed to treatments for 24 h before the MTT, NRU, and LDH assays were performed.

The MTT assay measures the activity of mitochondrial reductases, which convert water-soluble MTT salt to a formazan that accumulates in viable cells. After treatment, 20 μL of the MTT reagent (5 mg/mL) dissolved in DPBS were added to wells and incubated for 2 h at 37 °C. Solutions were removed from wells, and 100 μL of DMSO was added to each well and gently mixed on a shaker to solubilize formazan crystals. Absorbance readings of control and treated wells were taken against a DMSO blank at 570 nm using an Biotek Synergy HTX multi-mode reader (Santa Clara, CA).

The NRU assay measures the uptake of neutral red dye, which accumulates within the lysosomes of viable cells. Following the exposure of cells to treatments, all medium was removed. A working solution of 40 μg of neutral red stock/mL of cell culture medium was prepared and incubated at 37 °C overnight to dissolve the neutral red. Cells were incubated with 150 μL of neutral red solution for 2 h. Cells were washed with PBS, and 150 μL of lysis buffer (50% EtOH/49% deionized H2O/1% acetic acid) was added to each well and gently mixed to achieve complete dissolution. Absorbance readings of wells were recorded at 540 nm using a Biotek Synergy HTX multi-mode reader.

The LDH assay measures lactate dehydrogenase released into the culture medium due to plasma membrane damage. Reagents and solutions were prepared using an in-house recipe developed by OPS Diagnostics (Lebanon, NJ). TRIS (200 mM; 22.2 g of Tris-HCl, 10.6 g of Tris-base, and 50 mM lithium lactate) at a pH of 8 was prepared in water. INT was dissolved in DMSO (33 mg/mL), PMS was dissolved in water (9 mg/mL), and NAD sodium salt was dissolved in water (3.7 mg/mL). The three reagents (INT, PMS, and NAD) were combined to make the INT/PMS/NAD solution. All reagents (50 μL) were added to empty wells, followed by 50 μL of medium from treated and control wells. Absorbance readings were recorded at 540 and 620 nm using a Biotek Synergy HTX multi-mode reader.

### Growth and Morphology Assays

Noninvasive cell growth and morphology analyses of live cells were performed using 10× and 20× phase contrast objectives in a BioStation CT using the automatic Z-focus.^40^ After attachment, BEAS-2B cells were treated with Puff EC fluids (0.1–10%) or with WS-23 (0.045–4.5 mg/mL) solutions dissolved in cell culture medium. Images were taken every 2 h for 48 h to collect time-lapse data for analysis. Evaluation of BEAS-2B growth and morphology was compared in control and treated groups using Nikon CL Quant software (Melville, NY).^40−42^ Data from the treated groups were normalized to untreated controls.

### Solubility of WS-23 and WS-3 in Water and Culture Medium

WS-23 was dissolved in molecular grade water or culture medium at concentrations of 0.45, 4.5, 7, or 9 mg/mL, and 500 μL of each solution were added to 48-well plates with a glass bead in each well to aid in focusing the liquid with a stereoscopic microscope. For WS-3, 0.02 mg/mL was dissolved in water and cell culture medium to confirm its reported solubility. Images were taken with a stereoscopic microscope, and the presence of residues was compared for both solvents.

### Statistical Analyses

For GC/MS data, data points are averages of measurements from fluids obtained from three devices. All values below the limit of quantification (LOQ) were excluded from the data. Cytotoxicity analyses were performed using three different cell passages, and each experiment was carried out at least three times. Data were statistically analyzed with a one-way analysis of variance (ANOVA). When significance was found (*p* < 0.05), each concentration was compared to the untreated control with Dunnett’s post-hoc test using GraphPad Prism software (San Diego, CA).

## Results

### Total Concentrations of Nicotine and Flavor Chemicals

**Figure 1:**
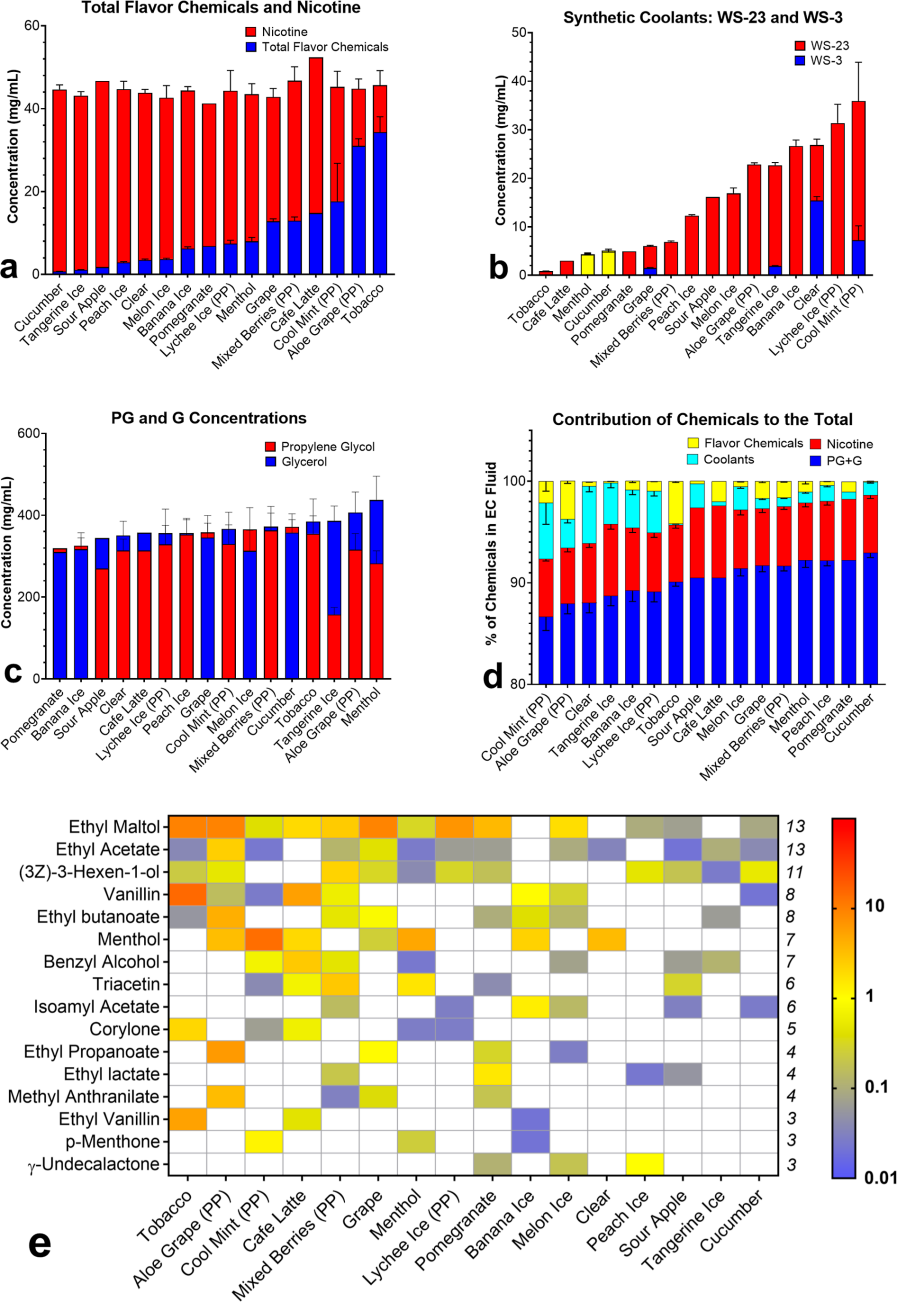
Chemical concentrations in Puff EC fluids. (a) Total flavor chemicals ranged from 0.7 to 34.3 mg/mL, and nicotine concentrations ranged from 41.2 to 52.3 mg/mL. (b) WS-3 and WS-23 concentrations ranged from 1.5 to 15.5 and 0.9 to 35.9 mg/mL, respectively. The *x*-axis is sorted by increasing total flavor chemical concentration and WS-23 in (a,b). Yellow bars in (b) indicate equal levels of synthetic coolants. (c) PG and G concentrations ranged from 158 to 371 and 310 to 437 mg/mL, respectively. (d) Percentage of each chemical class of chemicals in Puff products: flavor chemicals = 0.1–4.2%, synthetic coolants = 0.1−5.6%, nicotine = 5.5–7.1%, and solvents = 86.7–92.9%. (e) Heat map of individual flavor chemicals ordered on the *y*-axis according to the frequency of occurrence of dominant flavor chemicals. Products are ranked according to decreasing total weight (mg/mL) of the flavor chemicals on the *x*-axis from left to right. “PP” on the flavor name on the *x*-axis indicates “Puff Plus” products. Graphs show the means ± the standard deviation of three independent measurements (*n* = 3), except for “Sour Apple,” “Pomegranate,” and “Café Latte,” which are each based on one measurement.

Based on flavor names, Puff ECs were grouped into five categories: tobacco, fruity, berries, menthol, and unflavored. The concentrations of nicotine, flavor chemicals, synthetic coolants, and solvents were analyzed (Figure 1). The average nicotine concentration in disposable Puff devices (44.8 mg/mL ± 2.5 SD) was lower than that in previously evaluated JUUL pods (61 mg/mL), but higher than that in the cartomizer and refill fluids we have examined^28^ (Figure 1a). The total concentration of flavor chemicals in Puff fluids was highly variable and ranged from 0.7 (Cucumber) to 34.3 (Tobacco) mg/mL (Figure 1a). Fruit-flavored products were highly variable in total concentrations and dominant chemicals (>1 mg/mL). Seven flavor chemicals, including ethyl maltol and ethyl acetate in Aloe Grape, accounted for 80% of the sum of flavor chemicals. Minty flavored Puff ECs contained two dominant flavor chemicals: menthol and *p*-Menthone in “Cool Mint” and triacetin in “Menthol.” Although “Lychee Ice” and “Melon Ice” contained only ethyl maltol as the dominant flavor chemical, “Peach Ice” and “Clear” contained γ-undecalactone and menthol, respectively.

### Synthetic Coolants: WS-3 and WS-23

WS-3 and WS-23 were identified and quantified in both “ice” and “nonice” flavored Puff EC fluids (Figure 1b). WS-23 was present in all 16 products at concentrations ranging from 0.8 mg/mL in “Tobacco” to 45.1 mg/mL in “Cool Mint.” The levels of both synthetic coolants in “Cucumber” and “Menthol” were similar (5.1 and 4.3 mg/mL, respectively) and are shown using yellow bars in Figure 1b. WS-3 concentrations in 6/16 products were generally lower than WS-23 ranging from 1.5 mg/mL in “Tangerine Ice” to 16.4 mg/mL in “Clear” (Figure 1b). The concentrations of WS-3 in “Banana Ice,” “Mixed Berries,” and “Café Latte” were below the LOQ (0.02 mg/mL). The combined concentrations of WS-3 and WS-23 in products that contained both synthetic coolants ranged from 0.9 to 55.8 mg/mL.

EC products purchased and analyzed between 2016 and 2019 were re-evaluated to identify and estimate the concentrations of WS-3 and WS-23 in cartomizers, pods, and refill fluids (Table S1). Out of over 600 EC samples analyzed in our lab, both synthetic coolants were found in 13 products: WS-3 (*n* = 5) and WS-23 (*n* = 8) (Table S3). The concentrations of the synthetic coolants ranged from 0.2 to 1.7 mg/mL for WS-3 and 0.1 to 3.9 mg/mL for WS-23. Triethyl citrate was more frequently found in refill fluids at elevated levels and ranged from 0.05 to 11.5 mg/mL (Table S3).

### PG and Glycerol Concentrations

All Puff EC fluids contained PG and glycerol (G). The concentrations ranged from 158 to 371 and 310 to 437 mg of solvent/mL of undiluted Puff EC fluid for PG and G, respectively (Figure 1c). The sum of both solvents in Puff ECs ranged from 544 to 740 mg/mL. The percentage ratio of PG: G was approximately 30:70 in one product, 40:60 in three products, and 50:50 in 12 products (Figure 1c).

### Contributions of Chemicals to the Total Sum of Chemicals in Each Product

Chemicals in Puff ECs were grouped into four categories: nicotine, synthetic coolants (WS-3 and WS-23), flavor chemicals, and solvents (PG and G) (Figure 1d), and the percentage contribution of each group to the total sum of chemicals was calculated. Nicotine accounted for 5% of the total content in “Aloe Grape” to 7% in “Tangerine Ice,” “Sour Apple,” and “Café Latte.” The remaining 12 products contained 6% nicotine (Figure 1d). Synthetic coolant contribution to the total chemicals ranged from 0.1% in “Tobacco” to 6% in “Cool Mint” and “Clear” (unflavored product). In 75% of the products, synthetic coolant concentration to the total content was greater than 1% (Figure 1d). Flavor chemicals contributed between 0.09 and 4.2%, with more than half of the products higher than 1%. Solvents accounted for the most chemicals ranging from 87% in “Cool Mint” to 93% in “Cucumber.”

### Individual Flavor Chemicals in Puff Bar Fluids

Seventy-one percent (129/181) of the chemicals on our target analyte list were identified in Puff EC fluids. Forty-two flavor chemicals detected below the LOQ are listed in Table S2. Further analysis was performed on 87 flavor chemicals above the LOQ (Figures 1e and S1). Except for “Sour Apple,” “Tangerine Ice,” and “Cucumber,” all Puff ECs had at least one dominant flavor chemical (>1 mg/mL) (Figure 1e). Ethyl maltol, menthol, vanillin, ethyl propionate, ethyl butanoate, triacetin, methyl anthranilate, and (3*Z*)-3-hexen-1-ol were present in at least two products at >1 mg/mL. *p*-Menthone, ethyl lactate, corylone, isoamyl acetate, benzyl alcohol, ethyl acetate, ethyl vanillin, and γ-undecalactone were present in one product at >1 mg/mL. The concentrations of dominant flavor chemicals varied between Puff EC flavors and ranged from 1 to 15 mg/mL. Ethyl maltol was >1 mg/mL in 50% of the products evaluated. Less dominant flavor chemicals (0.02–0.99 mg/mL) are shown in Figure S1. While the frequency of all chemicals detected ranged from 1 to 16, the total number of chemicals per product ranged from 4 to 40 (Figures 1e, S1 and Table S2).

Major and minor nontarget chemicals were investigated for all Puff EC flavors. Benzoic acid, acetic acid, 2-hydroxypropyl acetate, 1,2-propanediol-2-acetate, 2-hydroxypropane-1,3-diyl diacetate, and glycerol 1,2-diacetate were identified as major nontarget compounds (Table S3). Vanillin and ethyl vanillin PG and G acetals were present as minor nontarget compounds in products that contained ≥5 mg of each chemical/mL of fluid (Figure 1e and Table S3).

### Cytotoxicity of Puff EC Fluids

**Figure 2:**
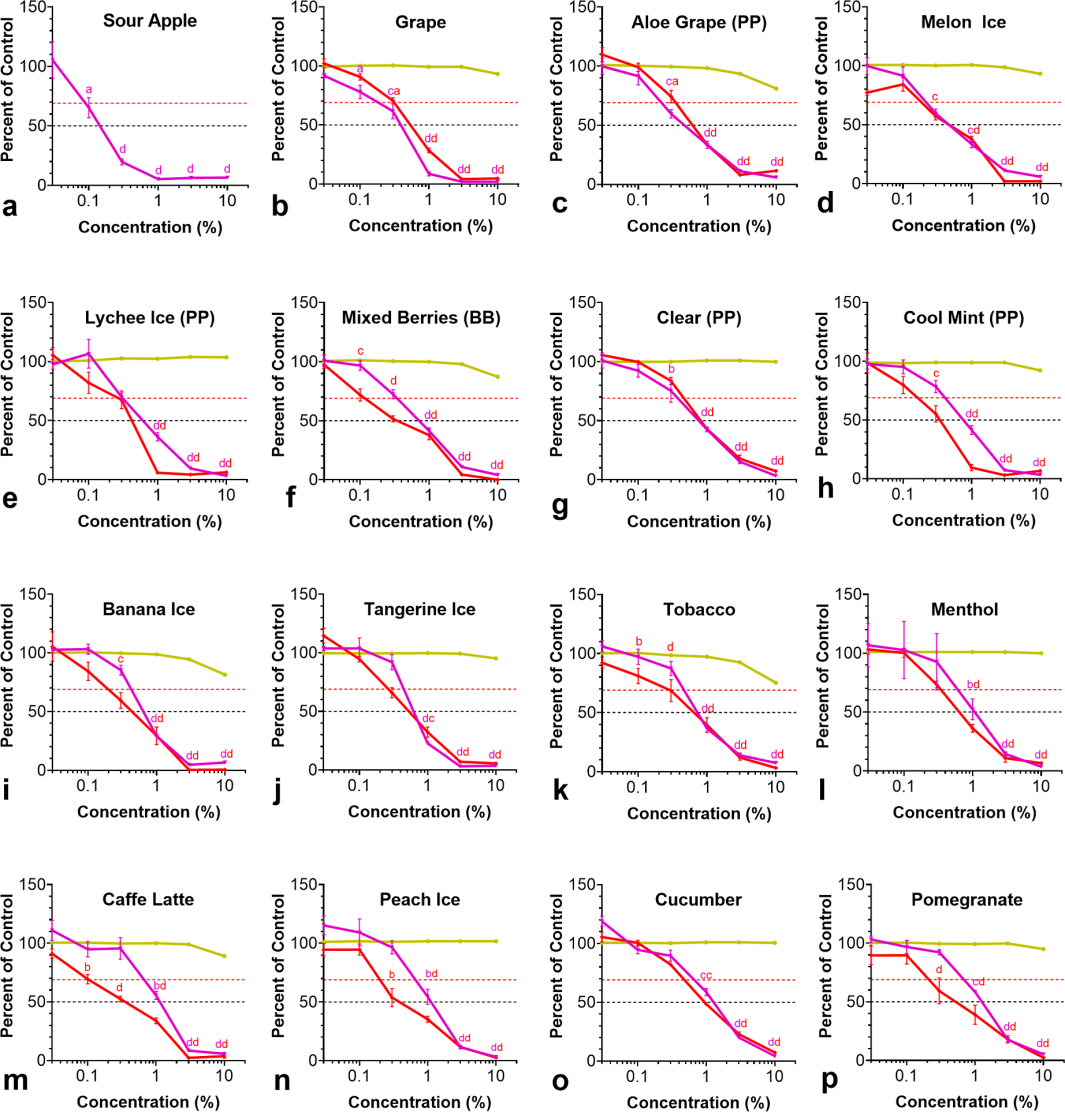
MTT, neutral red, and LDH assay concentration–response curves for BEAS-2B cells treated with Puff EC fluids. Purple line = MTT assay. Red line = neutral red assay. Yellow line = LDH assay. The *y*-axis shows the response of cells in each assay as a percentage of the untreated control. The concentrations tested were 0.03, 0.1, 0.3, 1, 3, and 10%. Each point is the mean ± standard error of the mean of at least three independent experiments. Red and black dotted lines on each graph represent IC_70_s and IC_50_s, respectively. For statistical significance, *a* = *p* < 0.05, *b* = *p* < 0.01, *c* = *p* < 0.001, and *d* = *p* < 0.0001.

Cytotoxicity of Puff EC fluids was evaluated with BEAS-2B cells using the MTT, NRU, and LDH assays (Figure 2 and Table 1). Products were considered cytotoxic if they had an effect of 30% less than the untreated control (IC_70_).^43^ Puff EC fluids were cytotoxic in the MTT and NRU assays, and IC_70_ and IC_50_ values were reached at fluid concentrations between 0.09–1.35 and 0.14–1.24%, respectively (Figure 2 and Table 1). Cell viability was evaluated using the LDH assay, and no significant effects were observed (Figure 2a–p).

**Table 1:** IC_50s_ and IC_70s_ for BEAS-2B Cells Treated with Puff EC Fluids^a^

	MTT (%)		NRU (%)		LDH (%)	
refill fluids	IC_50_	IC_70_	IC_50_	IC_70_	IC_50_	IC_70_
Sour Apple^b^	0.15	0.09	-	-	-	-
Grape	0.33	0.18	0.64	0.35	>10	>10
Aloe Grape (PP)	0.51	0.22	0.41	0.19	>10	>10
Melon Ice	0.51	0.21	0.38	0.36	>10	>10
Lychee Ice (PP)	0.64	0.31	0.35	0.12	>10	>10
Mixed Berry (PP)	0.72	0.35	0.90	0.47	>10	>10
Clear	0.77	0.38	0.31	0.17	>10	>10
Cool Mint (PP)	0.75	0.41	0.42	0.20	>10	>10
Banana Ice	0.68	0.42	0.55	0.26	>10	>10
Tangerine Ice	0.67	0.44	0.57	0.30	>10	>10
Tobacco	0.80	0.46	0.67	0.34	>10	>10
Menthol	1.08	0.61	0.32	0.10	>10	>10
Café Latte	1.10	0.66	0.48	0.20	>10	>10
Peach Ice	1.11	0.66	1.04	0.49	>10	>10
Cucumber	1.24	0.67	0.55	0.21	>10	>10
Pomegranate	1.23	0.68	0.53	0.32	>10	>10

a The highest concentration tested was 1% of the EC refill fluids.

b Sour Apple was not evaluated for NRU and LDH.

### Relationship between Chemical Concentrations and Cytotoxicity

**Figure 3:**
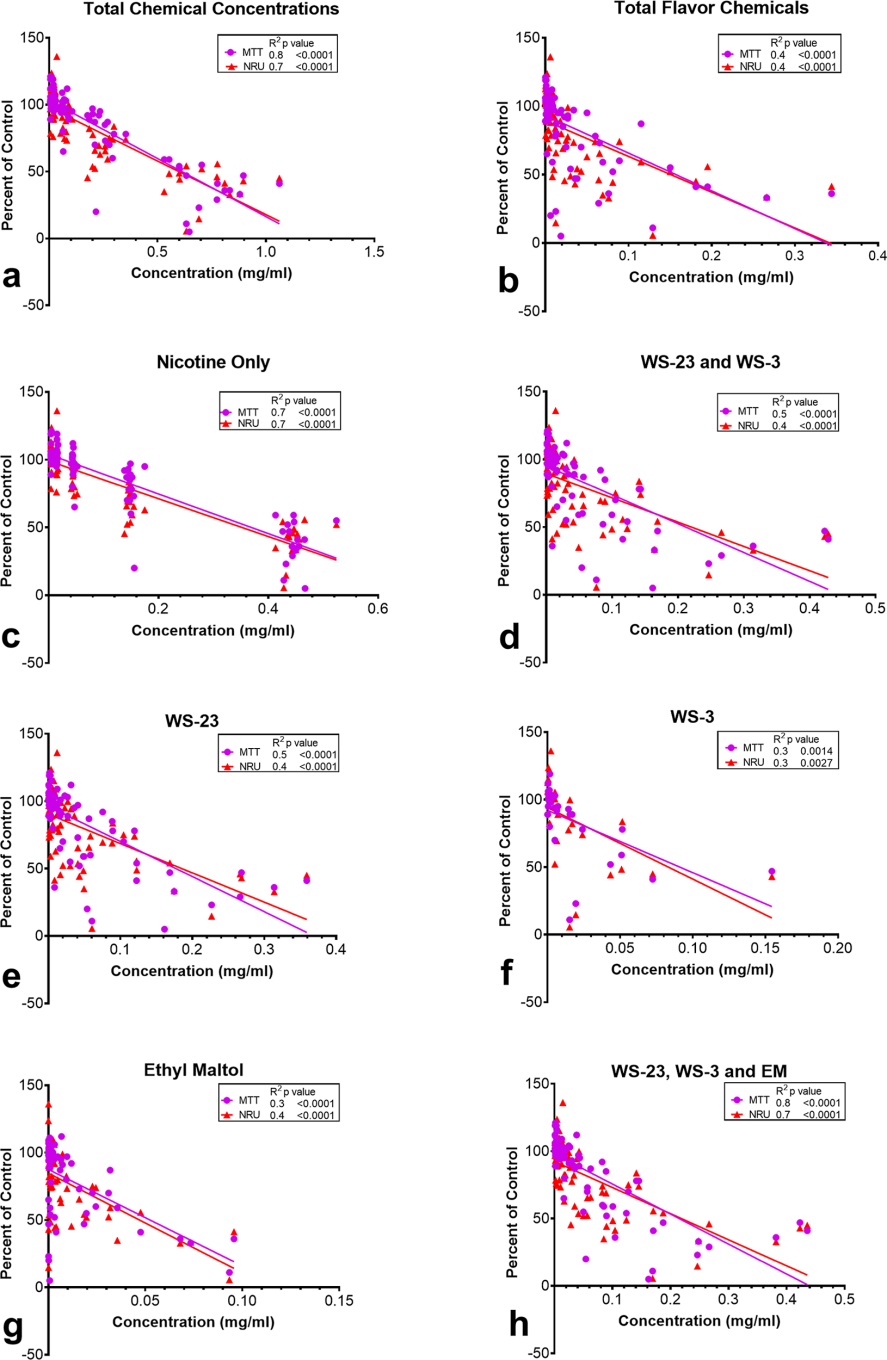
Relationship between the toxicity of Puff EC fluids in the MTT assay and the chemical concentrations of nicotine, WS-3, WS-23, and ethyl maltol in the Puff fluids. Linear regression analysis for cytotoxicity in the MTT assays (*y*-axis, expressed as a percentage of the untreated control) vs the concentrations of (a) total chemicals, (b) total flavor chemicals, (c) nicotine only, (d) WS-3 and WS-23, (e) WS-23 only, (f) WS-3 only, (g) ethyl maltol, and (h) synthetic coolants and ethyl maltol. Toxicity was strongly correlated (*R*^2^ ≥ 0.5) with the total chemicals, nicotine only, synthetic coolants, WS-23, and synthetic coolants and ethyl maltol. Total flavor chemicals, WS-3, and ethyl maltol were moderately correlated with toxicity (*R*^2^ < 0.5). All correlations were significant (*p* < 0.05). Linear regression analyses for toxicity vs other dominant flavor chemicals are shown in Figure S2.

Linear regressions were performed to determine the contributions of nicotine, flavor chemicals, and synthetic coolants to the cytotoxicity observed with Puff EC fluids (Figure 3). The chemical concentrations and cytotoxicity data for the 0.03–1% range were used to perform the regression analysis. Regression coefficients (*R*^2^) for concentration versus cytotoxicity were considered high (≥0.5), moderate (0.1–0.4), or low (≤0.1). High and moderate correlations were observed between cytotoxicity and concentrations of the total chemicals and flavor chemicals (Figure 3a,b). The regression analysis for nicotine only, a combination of synthetic coolants and WS-23, showed high and moderate correlations with significant *p*-values (Figure 3c–e). WS-3 and ethyl maltol concentrations were moderately correlated to cytotoxicity with significant *p*-values (Figure 3f,g). For products with both synthetic coolants and ethyl maltol, the relationship between cytotoxicity and concentration was high and statistically significant (Figure 3h). Regression analyses were performed for all other dominant flavor chemicals (Figure S2). The correlation coefficients ranged from moderate (Figure S2a–k) to no relationship (Figure S2l–o) with almost no significant *p*-values.

### Effect of WS-23 and Puff EC Fluids on Cell Growth and Morphology

**Figure 4:**
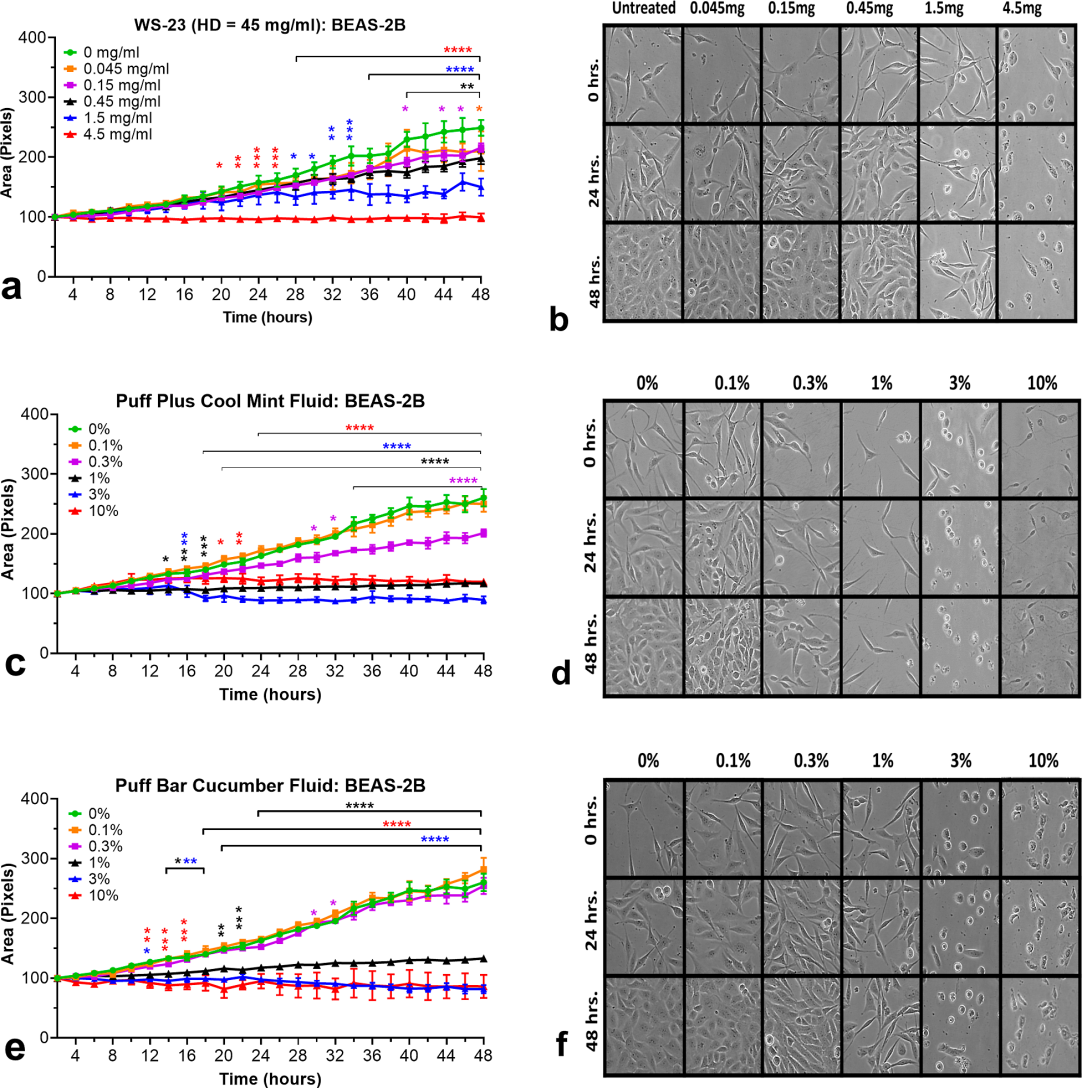
Effects of synthetic coolants and Puff EC fluids on cell growth and morphology in the live-cell imaging assay. Time-lapse imaging was performed with WS-23 (a,b), Puff Plus cool mint (c,d), and Puff Bar cucumber (e,f). In the cell growth experiments (a,c,e), the *x*-axis shows the duration of the experiment. The *y*-axis shows the mean of the percent increase in cell area (growth) over 48 h as determined using CL-Quant software. For cell morphology data (b,d,f), the *x*-axis shows the treatment concentration and the *y*-axis shows 24 h time intervals. Each point is the mean of at least three experiments ± the SEM. * = *p* < 0.05; ** = *p* < 0.01; *** = *p* < 0.001; **** = *p* < 0.0001.

Noninvasive analysis of BEAS-2B cell growth was performed using time-lapse images taken over 48 h (Figure 4a–f). The typical epithelial monolayer was observed for untreated control cells (Figure 4b,d,f). WS-23 significantly inhibited cell growth in a concentration-dependent manner (Figure 4a,b). Significance was observed as early as 20 h for cells treated with 10% (red lines), 28 h for 3% (blue lines), 40 h for 1 and 0.3%, and 48 h for 0.1% fluid solutions (Figure 4a,b). Cells appeared normal in all concentrations except in the 1.5 mg (3%) and 4.5 mg (10%) treatments, where the cells appeared elongated and rounded, respectively. Micrographs showing segmented images taken at 0, 24, and 48 h are presented in Figures S3a.

When Puff EC fluids with high levels of WS-23 (“Cool Mint”) and equal levels of WS-23 and WS-3 (“Cucumber”) were tested, varying effects were observed. The effects of the “Cool Mint” fluid (WS-23 = 45 mg/mL) and “Cucumber” fluid (equal concentrations of WS-3 and WS-23 = 5.1 mg/mL) were evaluated in a live-cell imaging assay. Cell growth was significantly affected starting at about 12 h for both treatments at concentrations of >1% (Figure 4c–f). BEAS-2B cells exposed to various concentrations of Puff Plus “Cool Mint” (Figure 4d) revealed elongated morphologies at 1%, rounded at 3%, or appeared fixed at 10% starting at the first time point and extending throughout the experiment. The morphologies observed with Puff Bar “Cucumber” fluid (Figure 4f) were either stressed and elongated (1%), rounded (3%), or fragmented (10%). Micrographs showing segmented images taken at 0, 24, and 48 h are presented in Figure S3b,c.

### Transfer Efficiency of Aerosolized Synthetic Coolants

**Figure 5:**
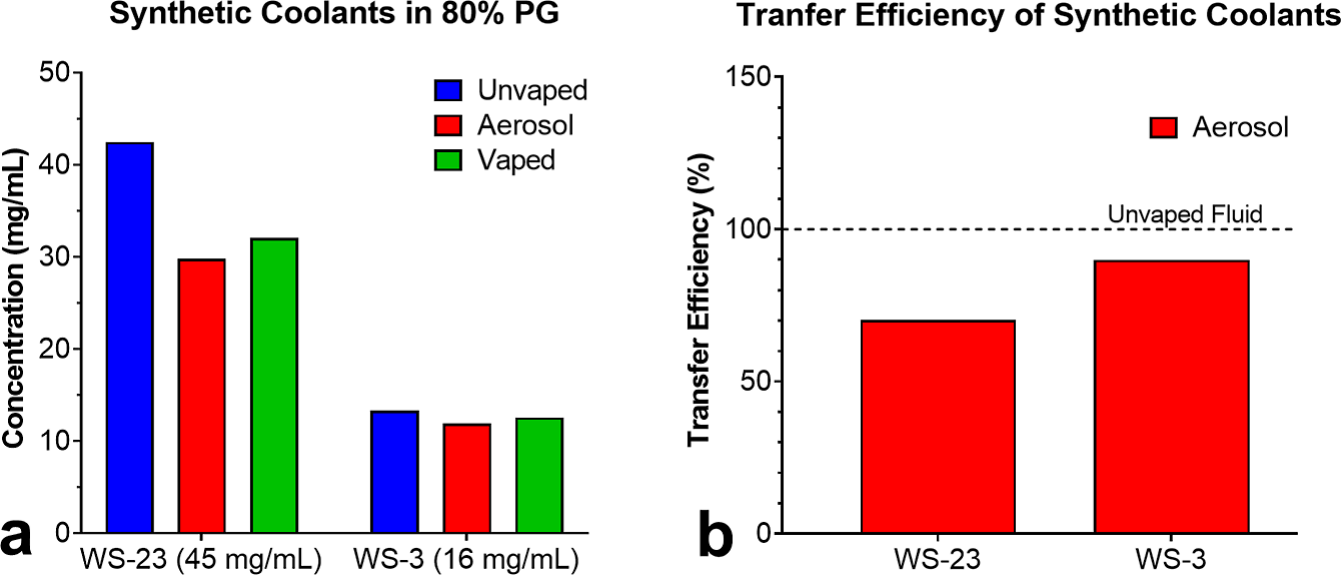
Synthetic coolants in lab-made refill fluids and their corresponding aerosols. (a) Concentrations of WS-23 and WS-3 in unvaped fluids, vaped fluids, and aerosols. (b) Transfer efficiency of WS-23 and WS-3 to aerosols. Aerosols were made using a fourth-generation Baton V2 open pod EC operating at 3.7 V/8.6 W. Each bar is a mean of two measurements.

Refill fluids made in-house using 80% PG, water, and authentic standards of each synthetic coolant were analyzed using GC/MS to identify and quantify chemicals in the fluids and corresponding aerosols (Figure 5). Generally, the transfer efficiency of aerosols produced with the Baton V2 pod device was high (Figure 5a). The mean of two experiments revealed that WS-23 transferred to an aerosol with 70% efficiency, while WS-3 transferred with 90% efficiency (Figure 5b). Puff Bar is also a low powered EC and likely has similar transfer efficiencies. Transfer efficiency can vary with many factors, including power, and may be higher in second- and third-generation ECs.

### MOE Evaluation for Synthetic Coolants

**Figure 6:**
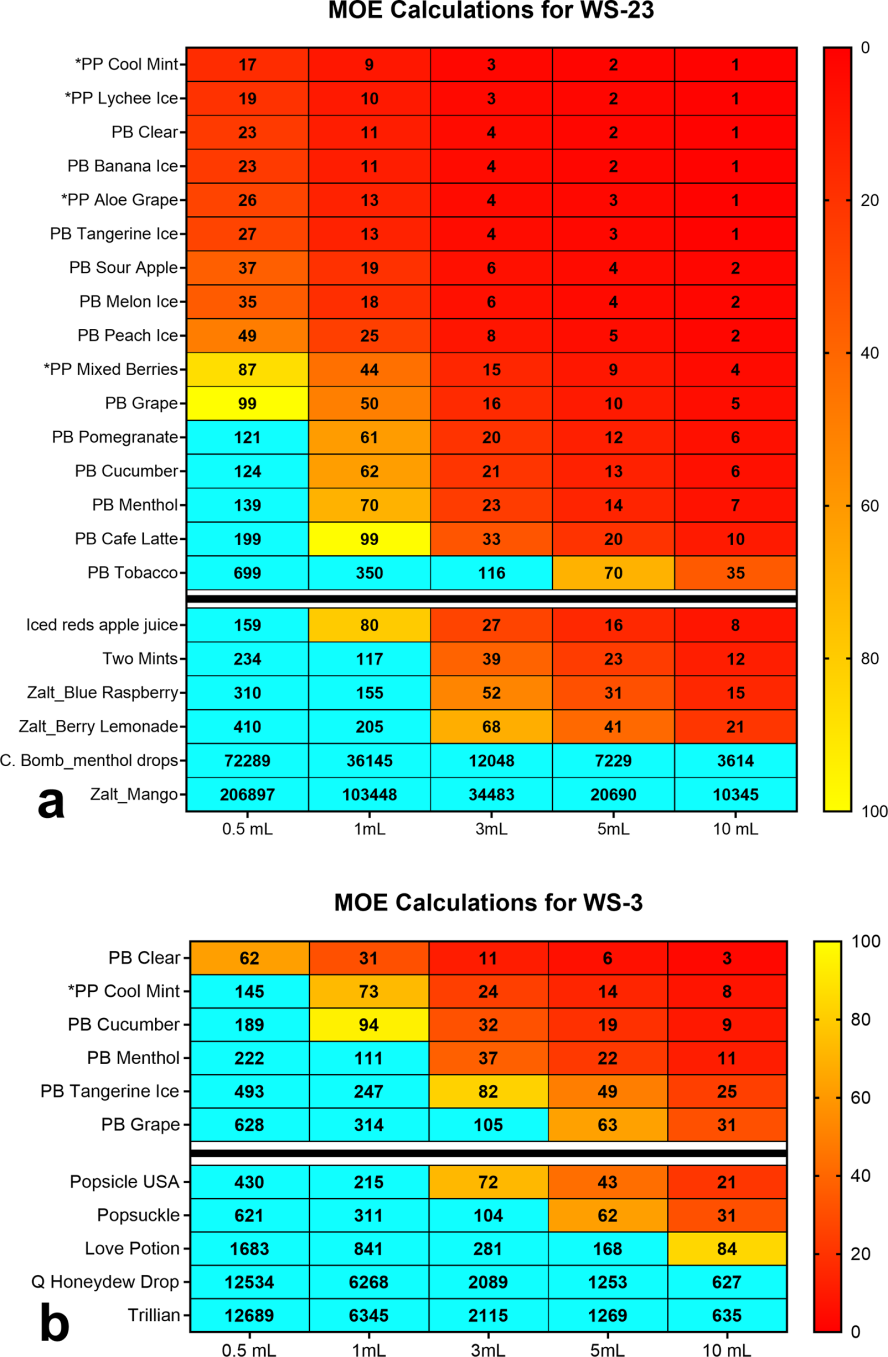
MOE for synthetic coolants in EC products. (a) WS-23 and (b) WS-3. MOEs below the threshold of 100 indicate a potential human health risk. The blue boxes are MOEs that were above the threshold of 100. EC products listed below the black horizontal bar indicate refill fluids and the Zalt brand of disposable ECs. “C” in “C. Bomb” in Figure 6a = cinnamon, “PP” = Puff Plus, and “PB” = Puff Bar.

The MOE, which aids risk assessors in prioritizing the potential exposure risk to food additives,^44,45^ was used to evaluate the potential risk from daily exposure to WS-3 and WS-23. The MOE approach considers a reference point (e.g., the NOAEL, no observed adverse effect level) from experimental data, an estimated daily exposure dose to the chemical or additive, and an average adult body weight of 60 kg. The daily consumption range of 0.5 mL (less than half the fluid in a Puff Bar device) to 15 mL, a high daily consumption for free-base nicotine EC fluids, was used. Using NOAEL values determined from orally administered WS-3 and WS-23 in rats, we calculated MOEs for WS-23 (NOAEL = 5 mg/kg/bw) and WS-3 (NOAEL = 8 mg/kg/bw)^46^ based on a 100% transfer from the EC fluid mixture into the aerosol. An MOE below the 100 threshold for a food additive is considered high risk requiring prioritization and mitigation by regulatory agencies. MOEs for WS-23 were <100 for all flavors except tobacco at 1 mL consumption per day (Figure 6a). For other nicotine-salt-based disposable devices and free-base nicotine fluids, daily consumption of 3 mL/day generated MOEs <100. In contrast, MOEs calculated for WS-3 were <100 in 5/6 products considering a 1 mL consumption per day (Figure 6b). Daily consumption of 3 mL/day generated WS-3 MOEs <100 in only 25% of the samples for other free-base nicotine fluids.

## Discussion

Our study investigated the chemicals in fluids from fourth-generation disposable Puff ECs and their toxicological effects. Over 100 chemicals, including nicotine and two synthetic coolants, were identified in 16 “ice” and “nonice” flavors. Nicotine concentrations in Puff fluids were generally lower than those previously reported in fourth-generation cartridge-based fluids.^27,28,47^ However, nicotine concentrations in Puff and JUUL^28^ were higher than those in free-base nicotine EC refill fluids.^48−52^ Two synthetic coolants (WS-3 and WS-23), often used in cosmetics, personal hygiene products, and edibles, were present in Puff EC fluids at concentrations higher than recommended for consumer products.^46^ The concentrations of WS-23 that inhibited mitochondrial reductases and cell growth were well below the concentrations in the Puff EC fluids. Concentration–response curves for toxic effects were significantly correlated with nicotine, ethyl maltol, and WS-23 concentrations. For most Puff ECs, the MOEs for the synthetic coolants were below the acceptable threshold of 100 for food additives, indicating a potential health risk.

Flavor chemicals in EC fluids and aerosols are frequently found in high concentrations and often account for a significant fraction of the total chemicals in EC products.^14,18^ We have previously categorized “dominant flavor chemicals” as those at concentrations ≥1 mg/mL.^17^ JUUL products generally had 0–1 dominant flavor chemical/product.^28^ In contrast, most (*n* = 13) Puff ECs had more than one dominant flavor chemical, and nine Puff e-cigarettes had two or more/products. Three Puff Bars (“Sour Apple,” “Tangerine Ice,” and “Cucumber”) did not have any dominant flavor chemicals. Puff Bar “Tobacco” contained the highest total flavor chemical concentration, with dominant chemicals being ethyl maltol, vanillin, corylone, and ethyl vanillin. In contrast, JUUL “Classic” and “Virginia Tobacco” did not have any dominant flavor chemicals,^28^ similar to other previously examined tobacco-flavored refill fluids.^15^ Although menthol was the dominant flavor chemical in “minty” Puff ECs, its concentration was two times higher in Puff Plus “Cool Mint” than in Puff Bar “Menthol”. *p*-Menthone, which may be added to enhance the minty flavor, was also dominant in Puff Plus “Cool Mint” and previously found at high levels in LiQua “Cool Menthol” refill fluids.^15^ Triacetin, a dominant flavor chemical in Puff Bar “Menthol,” may have been added to produce a fruity accent, or in the case of “Sour Apple,” it may have formed in part by a reaction between acetic acid and PG. In our prior studies, triacetin was not used frequently in American manufactured e-fluids.^14^ However, it was the most commonly used flavor chemical in a Russian brand (Ritchy LTD), distributed worldwide with high concentrations in fruity-flavored fluids (13–22.5 mg/mL) and a mint-flavored product without menthol (44.3 mg/mL).^15^ Ethyl maltol, a dominant and frequently used flavor chemical in multiple EC libraries,^15^ was in almost all Puff products at >1 mg/mL. In some previous studies, ethyl maltol was the most cytotoxic flavor chemical in the MTT assay, and its concentration was correlated with the cytotoxicity of JUUL and LiQua EC fluids.^15,18,28^

Some of the dominant flavor chemicals in Puff and JUUL ECs frequently appeared in high concentrations in our prior studies (e.g., menthol, ethyl maltol, benzyl alcohol, vanillin, and triacetin).^13,15,17^ Ethyl acetate and 3*Z*-3-hexen-1-ol were found in most Puff products, generally at concentrations <1 mg/mL. Ethyl acetate, which has low cytotoxicity in the MTT assay, was also present in most products in popular refill fluids.^18^

Both JUUL and Puff EC fluids contained benzoic acid, and two Puff flavors (“Sour Apple” and “Aloe Grape”) also had acetic acid. In addition, both 2-hydroxypropyl acetate and 1,2-propanediol-2-acetate were major nontarget chemicals in “Sour Apple,” “Aloe Grape,” “Tangerine Ice,” and “Peach Ice.” Both compounds are acetates of PG, which may be added as solvents or fruit flavorants, or form as reaction products between PG and acetic acid. Since acetic acid was a major nontarget chemical, it may be a reaction product.

Synthetic coolants were rarely used in earlier generations of EC products. When present, their concentrations were about 0.2 mg/mL in cartomizer fluids and 0.1–3.9 mg/mL in refill fluids, with WS-23 generally being higher than WS-3 (Table S1). WS-3 and WS-23 concentrations in Puff ECs sold in the USA were greater than those in JUUL pods sold in Europe or the USA.^19,53^ The synthetic coolants were present in all Puff ECs, while only two of eight JUUL flavors had synthetic coolants, which were significantly lower in concentration. WS-3 and WS-23 do not add flavor but impart a cooling sensation and were found in “ice” and “nonice” fruit, berries, and tobacco flavored Puff EC flavors. Concentrations of chemicals recently reported generally agreed with our data, except for menthol in “Cool Mint,” which was 22 times higher in our samples.^54^ This observation suggests batch-to-batch variations in Puff products. The constituents of EC fluids are rapidly evolving. In 2018, JUUL products contained very high nicotine concentrations combined with benzoic acid, which was not the case with refill fluids before the introduction of JUUL. Some Puff ECs contain synthetic coolant concentrations that are ∼450 times higher than the concentrations in JUUL (45.1 mg/mL in Puff Plus “Cool Mint” vs 0.1 mg/mL in JUUL “Classic Menthol”).^19^ The concentrations of nicotine, synthetic coolants, and flavor chemicals in Puff ECs are concerning and demonstrate the need for more attention to evolving EC constituents.

Fourth-generation JUUL pods are characterized by high concentrations of nicotine (∼61 mg/mL).^28^ Likewise, nicotine was relatively high in concentration in Puff products (40.6–52.4 mg/mL). In a related study, nicotine in Puff ECs ranged from 29.4 to 40.7 mg/mL,^52^ while another study found 83.4 mg/mL.^55^ Differences in reported concentrations for Puff ECs may be due to the methods used to quantify nicotine or variations in manufacturing different batches. In both studies, the reported nicotine concentrations are high relative to earlier generation products. PG/G ratios are similar to those reported previously for Puff products.^55^

Chemicals in EC products impair cell processes and induce inflammatory responses in multiple cell types.^16−23^ The concentrations of flavor chemicals and synthetic coolants in EC products are high enough to affect cell growth and morphology during acute exposure. In the current study, the cytotoxicity of Puff EC fluids in the MTT and NRU assays was significantly correlated with total chemical concentration and individual chemicals (nicotine, WS-3, WS-23, and ethyl maltol). The IC_50_s of fluids were lower when compared to similar flavors from JUUL in the MTT and NRU assays.^28^ We previously showed that the IC_50_ is reached for nicotine at 0.9 mg/mL in the MTT assay.^18^ The nicotine concentrations in Puff ECs are high enough to contribute to the toxicity of the fluids at the medium to high concentrations tested in the current study. Ethyl maltol, a frequently used dominant chemical,^15^ impairs the activity of mitochondrial reductases in BEAS-2B and mouse neural stem cells, with IC_50_s of 0.06 and 0.03 mg/mL in the MTT assay, respectively. The concentrations of ethyl maltol in Puff EC fluids are well above the IC_50_s reported previously.^18^ Both synthetic coolants in Puff ECs were evaluated for cytotoxicity, and WS-23 had a significant effect on mitochondrial metabolism at concentrations 90 times lower than those in Puff EC fluids (IC_50_ = 1 mg/mL). Our live-cell imaging analysis shows that WS-23 significantly affected cell growth and morphology shortly after the onset of treatment.

**Figure 7:**
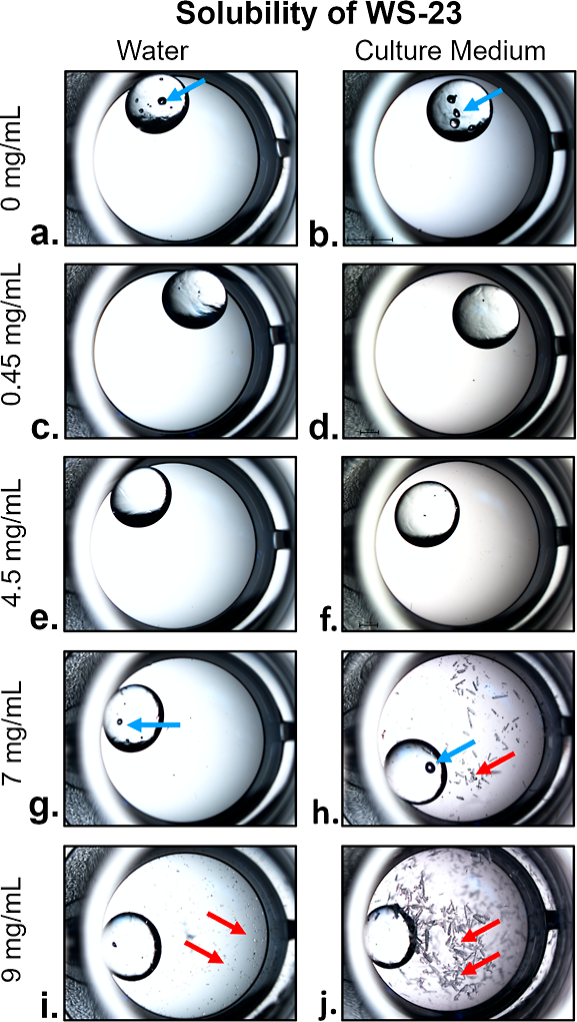
Stereoscopic microscopy images of droplets of water or culture medium containing various concentrations of WS-23 to show solubility (a–j). Both 0.45 and 4.5 mg/mL of WS-23 were soluble in water and culture medium (c–f). WS-23 (7 mg/mL) was soluble in 1 mL of water but not in BEAS-2B medium (g,h). Precipitates were present in both water (red arrows) and the culture medium containing 9 mg/mL (i,j). Blue arrows show air bubbles within the glass beads. The highest concentration used in our study was 4.5 mg/mL. The solubility of WS-3 and its toxicity at the reported 0.02 mg/mL concentration is shown in Figure S4.

There are conflicting reports on websites regarding the solubility of WS-23. PubChem and the Food and Agriculture Organization (FAO) of the United Nations report it is insoluble in water.^56,57^ The Good Scents Company and ChemHub websites report its solubility to be 0.45 mg/mL in water.^58,59^ In contrast, European and Chinese websites^60−62^ have reported the solubility of WS-23 to be ∼7 mg/mL, which is higher than the highest concentration we tested (4.5 mg/mL). To verify that WS-23 was dissolved at 4.5 mg/mL in our experiments, we tested its solubility in water and BEAS-2B culture medium at various concentrations (Figure 7). At 4.5 mg/mL, WS-23 was completely dissolved in water and culture medium (Figure 7c–f). At 7 mg/mL, WS-23 was soluble in water but not in culture medium (Figure 7g,h). At 9 mg/mL, a concentration above all reported solubilities, the chemical was partially soluble in water and insoluble in culture medium (Figure 7i,j). These data show that WS-23 was completely dissolved in our experiments at the highest concentration tested and further show that its reported solubility is incorrect on some websites.

Menthol and structurally related synthetic coolants such as WS-3 activate the TRPM8 channels located on cells, allowing ion influx and creating a cooling sensation, followed by activation of downstream inflammatory responses.^38^ WS-23 differs structurally from menthol yet imparts a cooling sensation. However, the lower potency of WS-23 to activate TRPM8 channels compared to menthol^63−65^ may indicate that other targets, including promiscuous TRP channels outside the M8 subfamily, may be involved in its effects on cells. Since these synthetic coolants, such as flavor chemicals, were not originally intended for use in inhalable products, minimal data exist on their adverse effect in humans after inhalation. A recent rat inhalation study found no significant effects of WS-23 on body weight, food consumption, and relative organ weights after a 4 h acute exposure and a 14 day observation period.^66^ In the same study, a 28 day subacute exposure followed by 28 days of recovery found no significant differences in body weight, food consumption, blood parameters, serum biochemistry, urine, pulmonary function, organ weight, and bronchoalveolar lavage fluid.^66^ However, the high dose used in the rat study (342.85 mg/m^3^) was one-eighth the concentration (2813 mg/m^3^) calculated for air exposure based on the highest concentration of WS-23 (45.1 mg/mL) in our study (assuming a 40 mL puff, 2.5 mg/puff, an aerosol density of 1 g/mL and WS-23 concentration). The concentration in the rat study may not have been sufficient to produce an effect, and/or the chosen endpoints may not have been affected. Similar animal exposure experiments using higher doses would be helpful.

Flavor chemicals are used in EC products at levels that exceed concentrations in other consumer products.^15,19^ Although these flavor chemicals are designated “Generally Regarded As Safe” (GRAS) for ingestion, the Flavor Extract Manufacturers Association (FEMA) does not endorse their use for inhalation.^67^ The concentrations of dominant flavor chemicals in Puff fluids were generally higher than those in edible products, except for ethyl vanillin in imitation vanilla extracts, which are diluted before use (Tables S4 and S5).^68−72^ Ethyl maltol, which imparts a sweet flavor, is frequently used at high concentrations in EC products.^13,14,17,19^ In edibles (e.g., beverages, candy, chewing gum, ice cream, and baked goods) and cosmetics (e.g., soaps, detergent, lotions, and perfume products), it is recommended that ethyl maltol concentrations do not exceed 0.4%.^68−73^ However, ethyl maltol in Puff fluids ranged from 0.007 to 0.99% and exceeded ingestible concentrations in 77% of the products when present. Ethyl maltol and some other flavor chemicals (e.g., ethyl vanillin and γ-decalactone) increase free radical formation in EC aerosols^74^ and contribute to the toxicity of EC fluids.^15,17,18^

Like flavor chemicals, synthetic coolants are designated GRAS and used in edible and skincare products.^71,72^ Even though their safety designation does not apply to inhalation, they have been used in tobacco products at 263–2300 ng/stick^75^ concentrations. The evolution of EC products has seen increased levels of synthetic coolants, especially with fourth-generation disposable products. WS-23 is used at 0.0008–0.3% in beverages, hard candy, confectionaries, and chewing gums.^71^ However, in Puff ECs, concentrations ranged from 0.08 to 4.51%. WS-3, another popular synthetic coolant, was found in fewer Puff ECs (38%) at 0.14–1.64% concentrations, exceeding maximum levels regarded as safe in beverages, ice creams, confectioneries, candy, and chewing gum (range = 0.001–0.12%).^72^ In the current study, the concentrations of synthetic coolants were up to thousands of times higher than in edible products and toxic in in vitro assays at concentrations lower than those found in Puff fluids.^18^ Consumers may be unwittingly exposed to high levels of synthetic coolants in “nonice” Puff ECs. Long-term studies with humans will be needed to fully understand the health effects of chronic inhalation of high concentrations of synthetic coolants.

Risk assessors use the MOE to evaluate carcinogenic risk or chemical safety based on predicted or estimated exposure levels. Since minimal data exist for inhalation exposures and toxicity, parameters based on oral administration of a chemical in experimental animals are often used.^76^ Nongenotoxic and noncarcinogenic chemical substances with MOEs less than 100 are generally considered a health risk. The concentrations of synthetic coolants in inhaled tobacco products exceed those in edible products. Calculated MOEs for WS-3 and WS-23 are well below 100 for almost all Puff products at 1 mL of fluid/day, thereby presenting a safety risk to consumers. Mint and “ice” flavored Puff ECs had the lowest MOEs, consistent with higher concentrations of synthetic coolants. Puff products that contained both synthetic coolants at levels that generated MOEs below the 100 thresholds would increase the exposure risks to users. Because the oral and inhalation toxicities are not always equivalent, route-to-route extrapolations routinely used by regulatory agencies^77,78^ may be required for a more realistic exposure model in humans. Considering the increased sensitivity of the respiratory tract to toxicants, the MOE values calculated for Puff ECs underestimate exposure.^77,78^ The Joint FAO/WHO (Food and Agricultural Organization of the United Nations/World Health Organization) Expert Committee on Food Additives concluded that further research is needed to assess the risk of synthetic coolants to humans.^76^

Future work should evaluate the use and concentrations of synthetic coolants in new EC products as they evolve. It would also be informative to examine exposure at the air-liquid interface using aerosolized synthetic coolants.

In summary, our data show that the fluid composition of ECs is evolving, with the most recent major change being the inclusion of high concentrations of synthetic coolants, which were toxic in our in vitro assays. The ban on flavored cartridge-based EC products caused a migration of adolescents and young adults from cartridge-based products such as JUUL to disposable ECs such as Puff, which is exempt from the flavor ban. These new disposable ECs, exemplified by the Puff brand studied here, have much higher concentrations of synthetic coolants than those found in JUUL. The high levels of nicotine, flavor chemicals, and synthetic coolants, which exceeded those used in other consumer products, raise a concern about the safety of Puff products. Product manufacturers are increasing the youth-attracting synthetic coolant content of ECs, while the inhalation risks remain unknown. This practice, in effect, represents a large, uncontrolled experiment in the lungs of youth and other consumers and highlights the need for regulation to protect public health.

## Supporting Information Available

Synthetic coolant concentrations in other EC products; flavor chemicals detected below the LOQ (0.02 mg/mL); flavor chemicals above the LOQ and <1 mg/mL; major and minor nontarget chemicals in Puff EC fluids; linear regression analysis for toxicity versus dominant flavor chemicals (continuation of Figure 3); micrographs showing segmented cells in the live cell imaging assay taken at 0, 24, and 48 h; MTT assay concentration–response curve and solubility of WS-3; flavor profiles of dominant chemicals in Puff EC fluids; and chemicals in EC fluids and average maximum levels (ppm) generally regarded as safe for their intended uses (PDF)
