# Supplementary material for: Disposable Puff Bar Electronic Cigarettes: Chemical Composition and Toxicity of E-liquids and a Synthetic Coolant
Source: Chem Res Toxicol. 2022 Jul 18;35(8):1344–58. doi: 10.1021/acs.chemrestox.1c00423 (PMC9382667; doi:10.1021/acs.chemrestox.1c00423)
Supplement: Supplementary file 1 — tx1c00423_si_001.pdf [file tx1c00423_si_001.pdf]

SUPPORTING INFORMATION

Disposable Puff Bar Electronic Cigarettes: Chemical Composition and Toxicity of E-liquids and a Synthetic Coolant

Esther E. Omaiye[†¶], *Wentai Luo*[‡§], *Kevin J. McWhirter*[‡], *James F. Pankow*[‡§] *and Prue Talbot*[¶*]

[†]Environmental Toxicology Graduate Program, University of California Riverside, California 92521, USA

[‡]Department of Civil and Environmental Engineering, Portland State University, Portland, Oregon 97201, USA

[§]Department of Chemistry, Portland State University. Portland, Oregon 97201, USA

[¶]Department of Molecular, Cell, and Systems Biology. University of California, Riverside, California 92521, USA

Corresponding Author
*Email: talbot@ucr.edu

TABLE OF CONTENTS

The Supporting Information is available free of charge

PAGE S3 Synthetic Coolant Concentrations in Other EC Products

PAGE S4 Flavor Chemicals Detected Below the Limit of Quantification (0.02 mg/mL)

PAGE S5 Flavor Chemicals Above the LOQ and < 1 mg/mL.

PAGE S6 Major and Minor Non-target Chemicals in Puff EC Fluids

PAGE S7 Linear regression analysis for toxicity versus dominant flavor chemicals (continuation of Figure 3).

PAGE S8 Micrographs showing segmented cells in the live cell imaging assay taken at 0, 24, and 48 hours

PAGE S9 Concentration-response curve of BEAS-2B cells treated with WS-3 in the MTT assay.

PAGE S10 Flavor Profiles of Dominant Chemicals in Puff EC Fluids

PAGE S11 Chemicals in EC Fluids and Average Maximum Levels (ppm) Generally Regarded as Safe for their Intended Uses

Table S1: Synthetic Coolant Concentrations in Other EC Products

	Refill Fluids	Pod Fluids	Cartomizer Fluids
**WS-3**			
Green Smoke Menthol			0.18
Green Smoke Menthol			0.20
Q Honeydew Drop	0.08 ± 0.014		
Love Potion	0.58 ± 0.101		
Popsuckle	1.65 ± 0.480		
**WS-23**			
Zalt Mango		<LOQ	
Cinnamon Bomb with menthol drops		<LOQ	
JUUL Cool Cucumber		0.03	
JUUL Classic Menthol		0.11 ± 0.02	
Zalt Berry Lemonade		1.46	
Zalt Blue Raspberry		1.94	
Two Mints	2.58 ± 0.24		
Iced reds apple juice	3.87 ± 0.86		

Table S2. Flavor Chemicals Detected Below the Limit of Quantification (0.02 mg/mL)

Flavor Chemical	CAS Number	Frequency[1]
Benzaldehyde	100-52-7	10
2-Acetylpyrrole	1072-83-9	9
Furfuryl alcohol	98-00-0	6
1,2-Dihydrolinalool	18479-51-1	5
6-Methyl-5-heptene-2-one	110-93-0	4
Ethyl Benzoate	93-89-0	4
Strawberry Glycidate B	77-83-8	4
Benzeneacetic acid, ethyl ester	101-97-3	3
α-Pinene	80-56-8 (7785-70-8)	3
β-Pinene	127-91-3	3
γ-Terpinene	99-85-4	3
2,5-dimethylpyrazine	123-32-0	2
Benzyl Benzoate	120-51-4	2
Butyl butyrate	109-21-7	2
Ethyl anthranilate	87-25-2	2
Ethyl Heptanoate	106-30-9	2
Ethyl octanoate	106-32-1	2
Fenchol	1632-73-1	2
Guaiacol (2-methoxyphenol)	90-05-1	2
Isoeugenol methyl ether	93-16-3	2
Methyl 2-methylbutyrate	868-57-5	2
p-Anisaldehyde	123-11-5	2
p-Cymene	99-87-6	2
Thymol	89-83-8	2
β-Myrcene	123-35-3	2
1,4-Cineol	470-67-7	1
1-Pentanol	71-41-0	1
2-Nonanone	821-55-6	1
Acetophenone	98-86-2	1
Ally hexanoate	123-68-2	1
Amyl Acetate	628-63-7	1
Benzyl Butyrate	103-37-7	1
cis-Linalool oxide	5989-33-3	1
Coumarin, 6-methyl	92-48-8	1
Estragole (4-allyanisole)	140-67-0	1
Geraniol Acetate	105-87-3	1
Isosafroeugenol	94-86-0	1
Methyl phenylacetate	101-41-7	1

[1]Frequency = number of times the flavor chemical appeared in at least one Puff Bar EC pod fluid

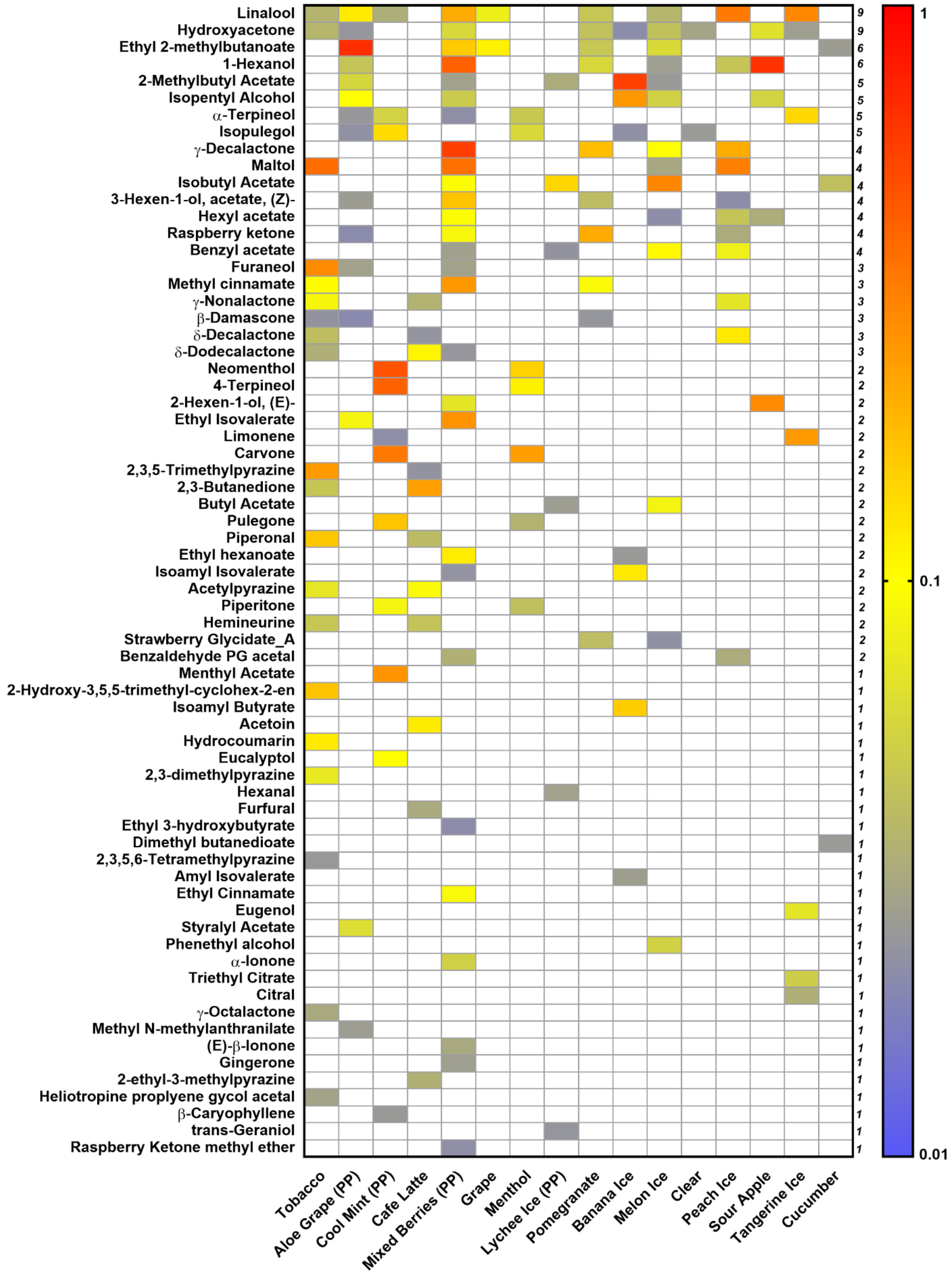

Figure S1. Heat map of 87 flavor chemicals above the LOQ with concentrations below 1 mg/mL in Puff EC fluids. Chemicals are ordered on the *y*-axis according to the frequency of occurrence of flavor chemicals from top to bottom. Products are ordered on the *x*-axis according to the total weight (mg/mL) of the flavor chemicals in each product with the highest concentration at the left. The color gradient on the right shows the concentrations of the flavor chemicals in the heat map.

Table S3. Major and Minor Non-target Chemicals in Puff EC Fluids

Sample	Major Non-targets	Minor Non-target
Sour Apple	Benzoic acid<br>Acetic acid<br>2-Hydroxypropyl acetate<br>1,2-Propanediol-2-acetate	
Aloe Grape (PP)	Benzoic acid<br>Acetic acid<br>2-Hydroxypropyl acetate<br>1,2-Propanediol-2-acetate	acetin (mixture of 2 isomers)<br>2-Hydroxypropane-1,3-diyl diacetate
Menthol	Benzoic acid<br>2-Hydroxypropane-1,3-diyl diacetate<br>Glycerol 1,2-diacetate	Neoisomenthol,<br>menthone isomer,<br>2-Hydroxypropyl acetate,<br>1,2-Propanediol-2-acetate
Pomegranate	Benzoic acid	Methyl (3-oxo-2-pentylcyclopentyl) acetate
Cafe Latte	Benzoic acid	vanillin PG and GL acetals
Tobacco	Benzoic acid	vanillin PG and GL acetals<br>ethyl vanillin PG and GL acetals
Melon Ice	Benzoic acid	(6Z)-Nonen-1-ol<br>2-(hydroxymethyl)-5-oxidanyl-2,3-dihydropyran-4-one
Cool Mint (PP)	Benzoic acid	Neoisomenthol,<br>menthone isomer
Tangerine Ice	Benzoic acid<br>2-Hydroxypropyl acetate<br>1,2-Propanediol-2-acetate	
Peach Ice	Benzoic acid<br>2-Hydroxypropyl acetate<br>1,2-Propanediol-2-acetate	
Clear	Benzoic acid	
Cucumber	Benzoic acid	
Grape	Benzoic acid	
Banana	Benzoic acid	
Lychee Ice (PP)	Benzoic acid	
Mixed Berries (PP)	Benzoic acid	

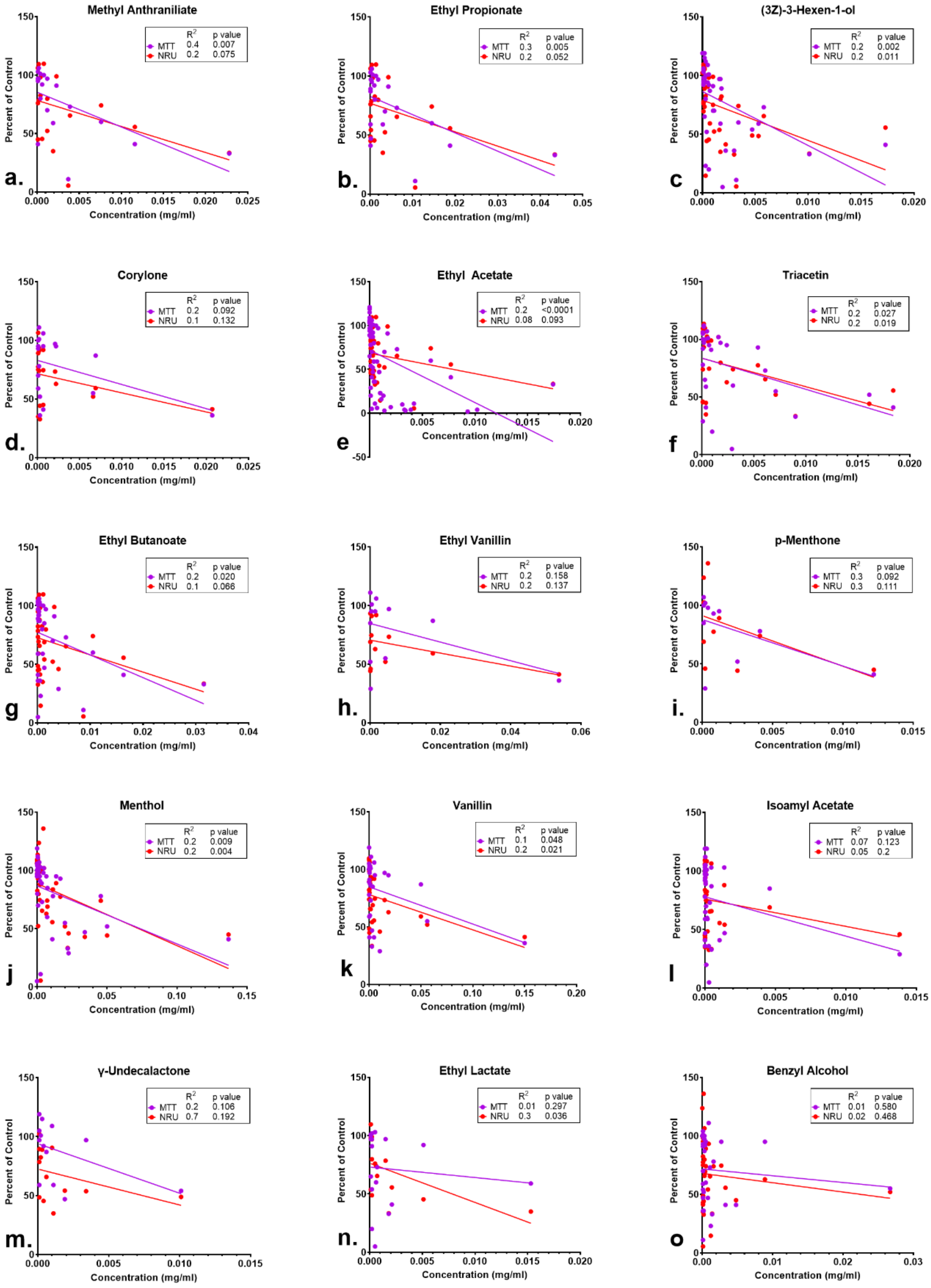

Figure S2. Regression analyses of dominant flavor chemicals not shown in the main text.

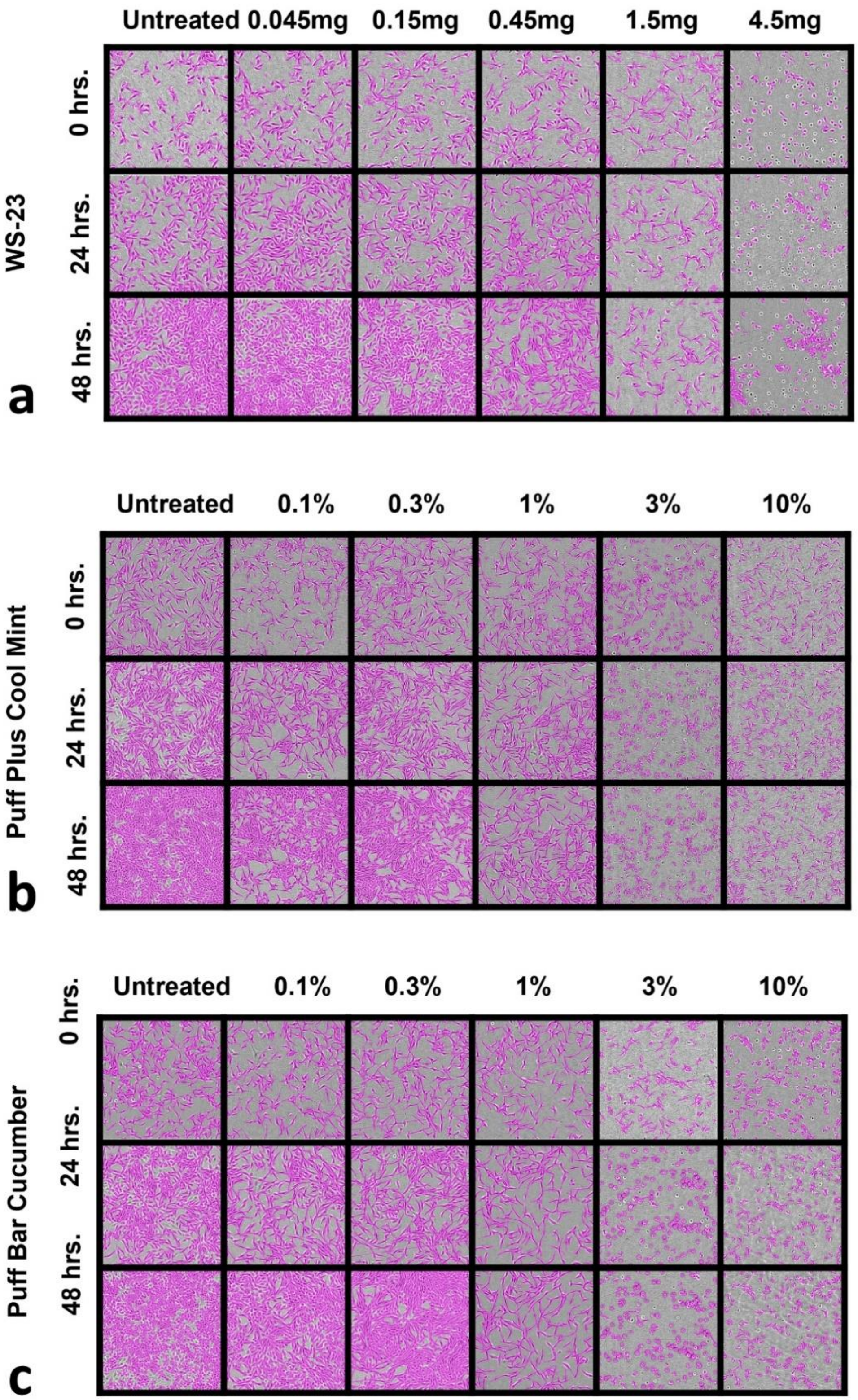

Figure S3. Micrographs showing segmented cells in the live cell imaging assay taken at 0, 24, and 48 hours.

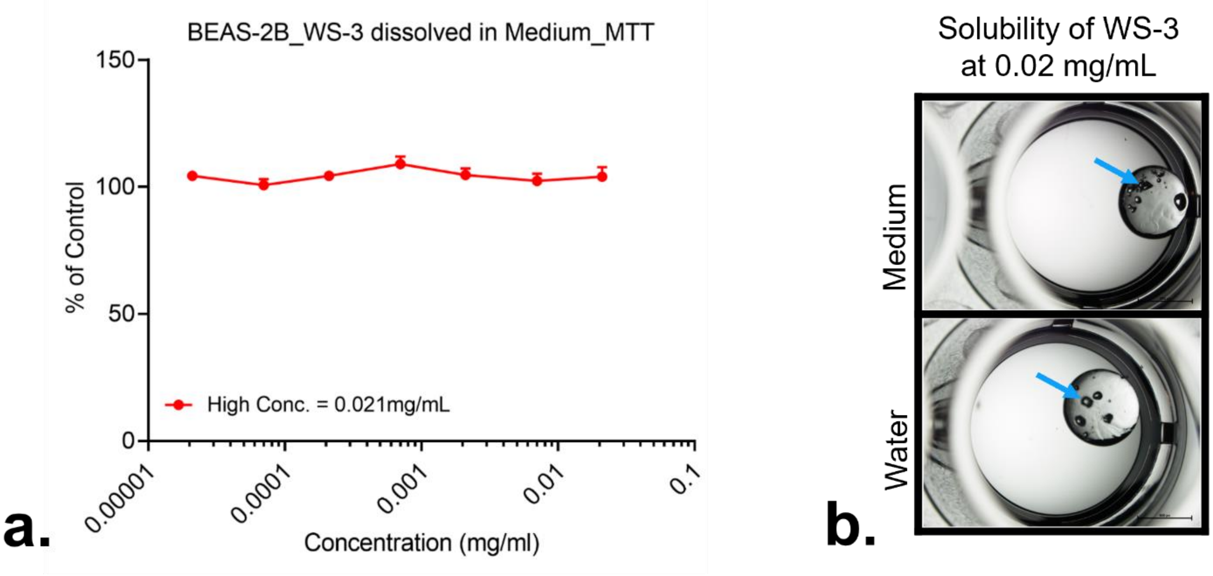

Figure S4. MTT assay concentration-response curve and solubility of WS-3. (a) MTT concentration-response curve for BEAS-2B cells treated with WS-3. The y-axis shows the response of cells as a percentage of the untreated control. Each point is the mean ± standard error of the mean of three independent experiments. (b) the solubility of WS-3 in culture medium and water at the highest concentration (0.02 mg/mL) used in the MTT assay. Each sample contains a glass bead to enable focusing on the liquid. Each bead has several black air bubbles (arrows). No precipitate is present in the medium or water solution containing WS-3 at 0.02 ug/mL.

Table S4: Flavor Profiles of Dominant Chemicals in Puff EC Fluids

Chemical	CAS #	FEMA#	FEMA Flavor Profile
Ethyl Maltol	4940-11-8	3487	Fruit
Ethyl Acetate	141-78-6	2414	Aromatic, Brandy, Contact Glue, Grape
(3Z)-3-Hexen-1-ol	928-96-1	2563	Grass, Green Fruit, Green Leaf, Herb, Unripe Banana
Vanillin	121-33-5	3107	Vanilla
Ethyl butyrate	105-54-4	2427	Apple, Butter, Cheese, Pineapple, Strawberry
Menthol	15356-70-4	2665	Mint, cool
Benzyl Alcohol	100-51-6	2137	Boiled Cherries, Moss, Roasted Bread, Rose
Triacetin	102-76-1	2007	Fruity (www.thegoodscentscompany.com)
Isoamyl Acetate	123-92-2	2055	Apple, Banana, Glue, Pear
Corylone	765-70-8	2700	Caramellic (www.thegoodscentscompany.com)
Ethyl Propanoate	105-37-3	2456	Apple, Pineapple, Rum, Strawberry
Ethyl lactate	97-64-3	2440	Cheese, Floral, Fruit, Pungent, Rubber
Methyl Anthranilate	134-20-3	2682	Flower, Honey, Peach
Ethyl Vanillin	121-32-4	2464	Floral
p-Menthone	10458-14-7	2667	Green, Fresh, Mint
γ-Undecalactone	104-67-6	3091	Apricot, Fruit
WS-23	51115-67-4	3804	Cooling
WS-3	39711-79-0	3455	Cooling

Table S5. Chemicals in EC Fluids and Average Maximum Levels (ppm) Generally Regarded as Safe for their Intended Uses

	Puff EC Fluids							Beverages			
**Chemical Name**	**Lowest Conc.**	**Highest Conc.**	**Chewing Gum**	**Hard Candy**	**Frozen Dairy, Ices**	**Baked Goods**	**Gelatins, Puddings**	**Non-Alcoholic**	**Alcoholic**	**Others**	**Reference**
Ethyl Maltol	70.6	9898	83	27.9	144	152	119	12.4	18.6	140	Oser and Ford, 1977[68]
Ethyl Acetate	20.4	2653.3	10000	7500	110	211	200	67	200	5000	Cohen et. al., 2020[69]
(3Z)-3-Hexen-1-ol	23.4	2411.5		5	3.7	5		1			Hall and Oser 1968[70]
Vanillin	21	16539.9	445	200	95	220	120	97	450	0	Cohen et al., 2020
Ethyl butanoate	44.6	4506.4	1400	98	44	93	54	28			Hall and Oser 1968
Menthol	251.2	18739.9	1100	400	68	130		35			Hall and Oser 1968
Benzyl Alcohol	23	2671.5	1200	47	160	220	45	15			Hall and Oser 1968
Triacetin	39.1	2814.6	4100	560	2000	1000		190			Hall and Oser 1968
Isoamyl Acetate	24.9	1538.9	2700	190	56	120	100	28			Hall and Oser 1968
Corylone	24.4	2109.6	15	18	5.6	13	14	11		30	Hall and Oser 1968
Ethyl Propanoate	27.8	6588.4	1100	78	29	110	15	7.7			Hall and Oser 1968
Ethyl lactate	22.1	1525.2	3100	28	17	71	8.3	5.4	1000	35	Hall and Oser 1968
Methyl Anthranilate	26.6	3423.4	2200	56	21	20	23	16	0.2		Hall and Oser 1968
Ethyl Vanillin	20.8	5860	110	65	47	63	74	20	100	28000	Hall and Oser 1968
p-Menthone	20.9	1488.1	8.7	71	33	52		7.7			Hall and Oser 1968
γ-Undecalactone	114	1059.8	90	11	3	7.1	7.5	4.4			Hall and Oser 1968
WS-3	1442.5	16356.4	1200	100	10		10	10	10	10	Newberne et. al., 1998[71]
WS-23	832.9	45143.8	3000	50					8	150	Smith et al., 1996[72]

Notes: Others include meat sauces, icings and toppings, soft candy, confectionery, frostings, syrups, jams and jellies, imitation vanilla, sweet sauces, fats and oils, meat products, poultry, milk products
